# Lack of acute phase response in the livers of mice exposed to diesel exhaust particles or carbon black by inhalation

**DOI:** 10.1186/1743-8977-6-12

**Published:** 2009-04-20

**Authors:** Anne T Saber, Sabina Halappanavar, Janne K Folkmann, Jette Bornholdt, Anne Mette Z Boisen, Peter Møller, Andrew Williams, Carole Yauk, Ulla Vogel, Steffen Loft, Håkan Wallin

**Affiliations:** 1 National Research Centre for the Working Environment, DK-2100 Copenhagen, Denmark; 2 Environmental Health Sciences and Research Bureau, Safe Environments Programme, Health Canada, Ottawa, Ontario, Canada; 3 Department of Environmental Health, University of Copenhagen, DK-1014 Copenhagen K, Denmark; 4 Department of Cellular and Molecular Medicine, University of Copenhagen, DK-2100 Copenhagen, Denmark; 5 National Food Institute, Technical University of Denmark, DK-2860 Søborg, Denmark; 6 Institute for Science, Systems, and Models, University of Roskilde, DK-4000 Roskilde, Denmark

## Abstract

**Background:**

Epidemiologic and animal studies have shown that particulate air pollution is associated with increased risk of lung and cardiovascular diseases. Although the exact mechanisms by which particles induce cardiovascular diseases are not known, studies suggest involvement of systemic acute phase responses, including C-reactive protein (CRP) and serum amyloid A (SAA) in humans. In this study we test the hypothesis that diesel exhaust particles (DEP) – or carbon black (CB)-induced lung inflammation initiates an acute phase response in the liver.

**Results:**

Mice were exposed to filtered air, 20 mg/m^3 ^DEP or CB by inhalation for 90 minutes/day for four consecutive days; we have previously shown that these mice exhibit pulmonary inflammation (Saber AT, Bornholdt J, Dybdahl M, Sharma AK, Loft S, Vogel U, Wallin H. Tumor necrosis factor is not required for particle-induced genotoxicity and pulmonary inflammation., Arch. Toxicol. 79 (2005) 177–182). As a positive control for the induction of an acute phase response, mice were exposed to 12.5 mg/kg of lipopolysaccharide (LPS) intraperitoneally. Quantitative real time RT-PCR was used to examine the hepatic mRNA expression of acute phase proteins, serum amyloid P (*Sap*) (the murine homologue of *Crp*) and *Saa1 *and *Saa3*. While significant increases in the hepatic expression of *Sap, Saa1 *and *Saa3 *were observed in response to LPS, their levels did not change in response to DEP or CB. In a comprehensive search for markers of an acute phase response, we analyzed liver tissue from these mice using high density DNA microarrays. Globally, 28 genes were found to be significantly differentially expressed in response to DEP or CB. The mRNA expression of three of the genes (serine (or cysteine) proteinase inhibitor, clade A, member 3C, apolipoprotein E and transmembrane emp24 domain containing 3) responded to both exposures. However, these changes were very subtle and were not confirmed by real time RT-PCR.

**Conclusion:**

Our findings collectively suggest that *Sap, Saa1 *and *Saa3 *are not induced in livers of mice exposed to DEP or CB. Despite pulmonary inflammation in these mice, global transcriptional profiling of liver did not reveal any hepatic response following exposure by inhalation.

## Background

Exposure to particulate air pollution is associated with cardiovascular morbidity and mortality [1-4]. However, the underlying mechanisms linking particulate air pollution and cardiovascular effects are unclear. It has been hypothesized that particle exposure may cause cardiovascular disease through particle-mediated lung inflammation leading to a systemic inflammatory reaction [5]. C-reactive protein (CRP) and serum amyloid A (SAA) are acute phase proteins produced in the liver in humans in response to inflammatory stimuli [6]. Humans exposed to ambient particulate matter (PM) have increased blood levels of CRP [5,7-9]. Epidemiological studies have shown associations between increased concentrations of SAA and CRP in plasma, and increased risk of cardiovascular diseases [10-12] and cancer [13].

Induction of an acute phase response has been reported in rodents exposed to particles in several studies. Fibrinogen and platelet activation, primary effectors of the acute phase response, has been observed in rodents following exposure to particles [14-16]. Recent studies have shown increased blood concentrations of CRP in rats after instillation [17] or inhalation [18] of PM. However, CRP is minimally induced in mice [19]. In contrast, SAA and serum amyloid P (SAP), the murine homologue to CRP, are strongly induced in mice following inflammatory stimuli. Acute-phase protein production is primarily regulated at the transcriptional level, although some post-transcriptional mechanisms may operate [20,21]. Therefore, mRNA expression levels of *Sap *and *Saa *may provide sensitive markers of systemic acute phase response in mice.

Diesel exhaust particles (DEP) are produced by incomplete combustion of diesel fuels. In urban settings, diesel exhaust is a prominent source of fine particles [22]. Carbon black (CB) is manufactured under controlled conditions for commercial use, primarily as a reinforcing agent in rubber and as black pigment in paints and printing inks. CB has been used in toxicological testing as a model particle for the carbonaceous core of DEP devoid of polycyclic aromatic hydrocarbons (PAHs) [23].

Inhalation and intratracheal instillation of DEP, CB or biofuel particles results in pulmonary mRNA expression of interleukin *(Il)-6 *in mice [24-26]. IL-6 is the chief stimulator for the production of most acute phase proteins [6]. However, evidence demonstrating the direct response of acute phase genes following pulmonary exposure to particulate air pollution is lacking. Our previous work investigated the induction of inflammation and DNA damage in mice exposed to DEP or CB particles by inhalation [25]. The mice responded with substantial pulmonary inflammation [25]. Given this positive response, in the present study we investigate potential markers of acute phase response in these mice.

## Results

### Hepatic expression of acute phase genes in mice exposed to lipopolysaccharide (LPS) by intraperitoneal injection

Exposure to LPS is known to result in an acute phase response. C57BL/6 mice were exposed to LPS as a positive control to confirm that we could detect an acute phase response with our approach. RT-PCR was used to quantify the expression of a few target genes in the liver, namely *Sap, Saa1 and Saa3*. Six hours after intraperitoneal (i.p.) injection of 12.5 mg/kg of LPS, a large increase in mRNA levels of *Sap *(2.5-fold, P < 0.05), *Saa1 *(4.5-fold, P < 0.001) and *Saa3 *(120-fold, P < 0.001) was observed in exposed animals compared to their matched controls administered saline i.p. (Table 1). The results confirm that the transcription of these three genes is up-regulated in the liver as part of the acute phase response.

**Table 1:** Hepatic mRNA expression levels of *Sap*, *Saa1 *and *Saa3*

Marker	0.9% NaCl i.p	LPS i.p.
*Sap*	0.00164 ± 0.00057	0.00408 ± 0.00055*
*Saa1*	0.00866 ± 0.00411	0.04081 ± 0.00423***
*Saa3*	0.00003 ± 0.00002	0.00360 ± 0.00087***

Relative hepatic mRNA expression levels of acute-phase markers in mice measured 6 h after i.p. injection of 12.5 mg/kg LPS or 0.9% NaCl

Messenger RNA expression levels were normalized to 18S rRNA. Mean ± SEM is shown (n = 4)

*P < 0.05 versus 0.9% NaCl

***P < 0.001 versus 0.9% NaCl

### Hepatic expression of acute phase genes in mice exposed to DEP or CB by inhalation

**Figure 1:**
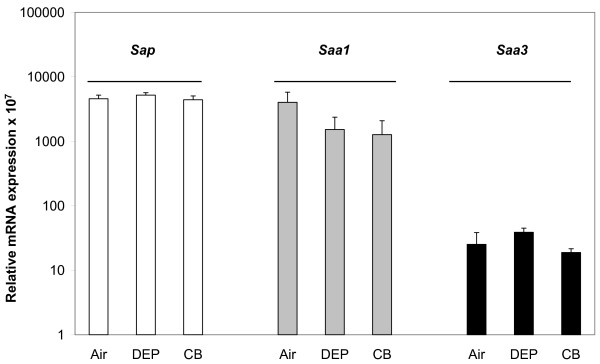
**Hepatic mRNA expression levels of *Sap*, *Saa1 *and *Saa3***. Relative *Sap *(white bars), *Saa1 *(grey bars) and *Saa3 *(black bars) mRNA expression levels in the liver of mice exposed to filtered air, DEP or CB by inhalation. Messenger RNA expression levels were normalized to 18S rRNA. Mean+SEM is shown. Each group consisted of 7–8 animals.

The mRNA expression of *Sap*, *Saa1 *and *Saa3 *(Figure 1) in livers from C57BL/6 mice exposed by inhalation to filtered air, 20 mg/m^3 ^DEP or CB for 90 min for 4 consecutive days was measured 1 h after the last exposure using RT-PCR. The expression of these genes was unaffected by exposure.

### SAA plasma concentration in mice exposed to DEP or CB by inhalation

The concentration of SAA in plasma from C57BL/6 mice exposed by inhalation to filtered air, 20 mg/m^3 ^DEP or CB for 90 min for 4 consecutive days was measured 1 h after the last exposure. The concentration of SAA in plasma was unaffected by exposure (data not shown).

### Microarray analysis of liver tissue from mice exposed to DEP or CB by inhalation

Changes in gene expression were analysed using Agilent Mouse Oligo Microarrays (G4121A). Statistically significant differential expression was defined as up-or down-regulation at the false-discovery-corrected level P < 0.05, compared with filtered air-exposed controls. Of 21,318 genes examined, 25 genes were affected by CB exposure and 6 were affected by DEP exposure compared to filtered air-exposed mice (Table 2). Complete DNA microarray data are available in NCBI gene expression and hybridization array data repository (GEO, ), accession number GSE11346. Three genes (*Serpina3c*, *Tmed3 *and *Apoe*) were down-regulated in response to both exposures. Exposure to CB and DEP caused a 1.9 and a 2-fold decrease in *Serpina3c*, a 1.7 and a 1.6-fold decrease in *Apoe*, and a 1.3 and 1.4-fold decrease in *Tmed3 *expression respectively. However, subsequent real-time PCR validation did not confirm the microarray analysis (data not shown). Transcriptional profiling with microarrays revealed no changes in the expression profiles of *Sap, Saa1 *and *Saa3*, confirming the lack of response measured using real-time RT-PCR following exposure to DEP and CB. Indeed, given the very small number of genes from the 21,318 on the microarray that exhibited p-values less than 0.05, it is possible that the majority of the genes in the list are false positives. It is clear that the exposure to DEP and CB produces remarkably few changes in liver gene expression at the time point examined, and the changes that are induced are likely to be very subtle.

**Table 2:** List of genes up or down regulated in the livers of mice exposed to CB or DEP by inhalation

**Systematic Name**	**Description (mus musculus mRNA)**	**CB**	**DEP**
NM_008458 *	Serine (or cysteine) proteinase inhibitor, clade A, member 3C (Serpina3c)	-1.89	-2.00
NM_009696 *	Apolipoprotein E (Apoe)	-1.69	-1.64
NM_025360 *	Transmembrane emp24 domain containing 3 (Tmed3)	-1.29	-1.37

NM_001011744 **	Olfactory receptor 105 (Olfr105)	1.35	1.36
NM_020569 **	Parkinson disease (autosomal recessive, early onset) 7 (Park7)	-1.31	-1.26
AK045923 **	Adult male corpora quadrigemina cDNA, RIKEN full-length enriched library	1.23	1.21
NM_026987 **	DEAH (Asp-Glu-Ala-His) box polypeptide 16 (Dhx16)	1.20	1.16
NM_008113 **	Rho GDP dissociation inhibitor (GDI) gamma (Arhgdig)	-1.24	-1.18
NM_011174 **	Proline rich protein HaeIII subfamily 1 (Prh1)	-1.38	-1.19
NM_146350 **	Olfactory receptor 1123 (Olfr1123)	-1.31	-1.21
NM_010324 **	Glutamate oxaloacetate transaminase 1, soluble (Got1)	-1.38	-1.24
NM_032541 **	Hepcidin antimicrobial peptide 1 (Hamp1)	-1.57	-1.31
NM_009997 **	cytochrome P450, family 2, subfamily a, polypeptide 4 (Cyp2a4)	-1.78	-1.47
NM_027853 **	RIKEN cDNA 0610006F02 gene (0610006F02Rik)	-1.69	-1.32
NM_022434 **	Cytochrome P450, family 4, subfamily f, polypeptide 14 (Cyp4f14)	-1.69	-1.25
AK078353 **	10 days neonate cerebellum cDNA, RIKEN full-length enriched library	1.29	1.11
NM_008281 **	Hepsin (Hpn)	-1.48	-1.21
NM_025363 **	RIKEN cDNA 1110001J03 gene (1110001J03Rik)	-1.23	-1.09
NM_175641 **	Latent transforming growth factor beta binding protein 4 (Ltbp4)	1.19	1.07
NM_177657 **	Hypothetical protein D630003M21 (D630003M21)	-1.27	-1.08
NM_177661 **	RIKEN cDNA C130079G13 gene (C130079G13Rik)	-1.32	-1.09
AK044171 **	10 days neonate cortex cDNA, RIKEN full-length enriched library	-1.19	-1,.6
NM_027318 **	Zinc finger, HIT domain containing 1 (Znhit1)	-1.23	-1.05
NM_008081 **	UDP-N-acetyl-alpha-D-galactosamine:(Galgt2)	-1.28	-1,.5
NM_026171 **	Nuclear VCP-like (Nvl)	1.39	1.01

NM_007506 ***	ATP synthase, H+ transporting, mitochondrial F0 complex, subunit c (subunit 9), isoform 1 (Atp5g1)	-1.18	-1.24
BC013546 ***	RIKEN cDNA 1810054G18 gene	-1.25	-1.32
BF531488 ***	BF531488 602091718F1 NCI_CGAP_Co24	-1.02	-1.23

* Significantly differentially expressed in both CB and DEP exposed group

** Significantly differentially expressed only in CB exposed group

*** Significantly differentially expressed only in DEP exposed group

Fold changes are over matched controls.

In addition to analysis of statistically significant differential expression, we ranked the genes according to fold change in response to the type of exposure (data not shown). This analysis demonstrates that overall gene expression was relatively unchanged in the livers. Within the list of differentially expressed genes, the largest was a halving of the mRNA expression of *Serpina3c*, but this was not confirmed by PCR.

## Discussion

Induction of the acute phase system is proposed to be one mechanism by which particulate exposure may affect the cardiovascular system [5]. This hypothesis is based primarily on observations in epidemiological studies showing an association between CRP and ambient air pollution [8,9]. In the present study, we investigated the utility of transcriptional changes in genes involved in acute phase response to predict possible adverse cardiac events in mice exposed to DEP or CB particles by inhalation.

CRP and SAA are acute phase response proteins synthesized by the liver during inflammatory reactions in response to IL-6. PM, when deposited in lung, stimulates production of cytokines such as IL-6 [18], which then pass through the blood stream to the liver and induce CRP and SAA production. We previously found considerable pulmonary inflammation in mice exposed to four consecutive doses of DEP or CB by inhalation. This was marked by a 2-fold increase in pulmonary *Il-6 *and Tumor necrosis factor (*Tnf*) mRNA expression in response to both DEP and CB inhalation, and a 4-fold increase in the percentage of neutrophilic granulocytes in the lung lining fluid in response to DEP inhalation [25]. However, analysis of the livers of these mice in the present study revealed no changes in mRNA expression of *Sap *or *Saa*. One potential explanation for the lack of *Sap *or *Saa *mRNA induction could be that DEP and CB mediate their effects through different pathways that involve other acute phase proteins.

To investigate this possibility, DNA microarrays were used to quantify global gene expression changes in liver tissue from the mice exposed to four consecutive doses of DEP or CB by inhalation. Microarray analysis revealed differential expression of modest magnitude for a limited number of genes. Three genes were down-regulated by both DEP and CB exposure: serine(or cysteine) proteinase inhibitor, clade A, member 3C (*Serpina3c*), apolipoprotein E (*Apoe*) and transmembrane emp24 domain containing 3 (*Tmed3*). However, these results could not be confirmed by real-time RT-PCR, suggesting that these are false positives, or extremely subtle changes that may not be biologically-relevant. Therefore, global transcriptional analysis demonstrates that the livers of these mice are surprisingly unresponsive to inhalation of DEP and CB. Gene expression profiling with microarrays also confirmed the lack of response measured for specific acute phase genes using RT-PCR.

Hepatic mRNA expression was investigated in this study because the liver is the primary site of mRNA and protein synthesis of acute phase proteins in response to inflammatory stimuli. However, most epidemiological evidence of a relationship between PM and induction of an acute phase response is based on concentrations of acute phase markers in the blood. To examine the possibility that lack of response in our study was due to the analysis of mRNA expression instead of protein concentrations, we also measured the plasma concentrations of SAA. Our findings were consistent with the lack of change in *Saa *expression; SAA protein concentrations were not affected by inhalation of DEP or CB.

We evaluated the hepatic acute phase response in mice which responded with increased inflammation after inhalation of particles on four consecutive days in a previous study [25]. Our study design was based on work by Peters et al. [27] demonstrating that the blood concentration of CRP was increased immediately in response to elevated levels of particulates during an air pollution episode in Europe in 1985, and a cumulative effect appeared to be present when interpreting 5 day means of exposure [27]. To our knowledge, no animal experiments have been published addressing the relationship between repeated particle exposures and the hepatic acute phase response. However, mice exposed to four repeated intraperitoneal injections of dimethylnitrosamine given on each of four consecutive days increased the hepatic mRNA expression of SAA and SAP similar to that of a chronic inflammatory state [28]. Based on this evidence, we chose to evaluate the systemic acute phase response after 4 days of dosing. However, since our findings show remarkably few changes both in global gene expression and targeted analysis of acute phase response genes at the selected time point, it is possible that a longer exposure period may cause a stronger global hepatic response to inhalation of DEP and CB.

## Conclusion

In conclusion, no systemic acute phase response was observed in mice following inhalation of particles at doses inducing substantial pulmonary inflammation. A search for response in other hepatic acute phase genes did not identify any promising candidates.

## Methods

### Particles

DEP were Standard Reference Material 2975 from the National Institute of Standards and Technology (NIST) (Gaithersburg, MD, USA). Carbon black particles (Printex F 90) were kindly donated by Degussa-Hüls, Frankfurt, Germany. As reported earlier, we determined the specific surface areas by multipoint BET (Brunauer, Emmett, and Teller) nitrogen adsorption (Micromeritics, Gemini 2375) [25]. The specific surface area was 295 m^2 ^g^-1 ^for CB and 90 m^2 ^g^-1 ^for DEP. NIST reported the specific area of DEP to be 91 m^2 ^g^-1^.

### Mice and exposure

We analyzed the hepatic acute phase response in livers from C57BL/6 mice that were part of a study investigating pulmonary inflammation and DNA damage. Data on pulmonary effects and DNA damage of the exposure to DEP and CB in these mice have been published previously [25]. The study design has been described earlier [25]. Briefly, particles were aerolized by using a microfeeder with dispersion nozzle (Fraunhofer Institut für Toxicologie und Aerosolforschung). 10–12 week old C57BL/6 mice (n = 7–8 per group) were exposed by inhalation for 90 min on four consecutive days of 20 mg/m^3 ^DEP or CB. Control mice were exposed similarly to filtered air. Mice were anaesthetized with Hypnorm/Dormicum 1 h after the last exposure and full blood was drawn from the heart before the lungs were lavaged. The blood was transferred to a tube containing K_2_EDTA and gently mixed. After centrifugation at 2000 g for 10 min, the supernatant was transferred to a new tube and stored at -80°C until further analysis.

As a positive control for acute phase response, we used livers from mice exposed to LPS as part of a previously published study [29]. Briefly, 8–9 week old C57BL/6 mice (n = 4 per group) received i.p. injections of 0 or 12.5 mg/kg LPS (Sigma-Aldrich Chemie; L8274-100 MG) in saline. Mice were anaesthetized in an inhalation chamber 6 h after the exposure with 4% isoflurane in 1:1 N_2_O/O_2 _. The anaesthesia was maintained with an inhalation mask containing 1% isoflurane in 1:1 N_2_O/O_2 _and the mice were killed by cervical dislocation. The livers were snapfrozen in liquid nitrogen and stored at -80°C.

The experiments were approved by the Danish "Animal Experimental Inspectorate" and carried out following their guidelines for ethical conduct and care when using animals in research.

### Quantitative PCR

#### Preparation of RNA and cDNA

Hepatic RNA from the C57BL/6 was prepared as described earlier [25]. cDNA was prepared from DNase treated RNA using TaqMan reverse transcription reagents (Applied Biosystems, USA) as recommended by the manufacturer.

#### Real-time RT-PCR

*Saa1*, *Saa3 *and *Sap *gene expression was determined using real-time RT-PCR with 18S RNA as the reference gene. Each sample was run in triplicate on the ABI PRISM 7700 sequence detector (PE Biosystems, Foster City, CA, USA). For *Saa1 (Mm00656927 gi) *and *Sap (Mm00488099 g1)*, TaqMan pre-developed reaction kits (Applied Biosystems, USA) were used. *Saa3 *primers and probes were designed with Primer Express (Applied Biosystems, Nærum, Denmark). The sequences of the *Saa3 *primers and probe were: Saa3forward: 5' GCC TGG GCT GCT AAA GTC AT 3', Saa3reverse: 5' TGC TCC ATG TCC CGT GAA C 3' and Saa3probe: 5' FAM-TCT GAA CAG CCT CTC TGG CAT CGC T-TAMRA 3'. The specificity of the probes was verified against the National Center for Biotechnology Information (NCBI) GeneBank. In all assays, TaqMan pre-developed mastermix (Applied Biosystems) was used. Target and 18S RNA levels were quantified in separate wells. The relative expression of the target gene was calculated by the comparative method 2^-ΔCt ^[30]. The average standard deviation on triplicates was 15%. The standard deviation on repeated measurements of the same sample (the control) in separate experiments was 25%, indicating that the day-to day variation of the assay was 25%. The probes and primers have been validated and the assay was quantitative over a 32- to 64-fold range. Messenger RNA measurements were excluded if the 18S content fell outside the range in which the PCR was found to be quantitative defined by the validation experiments. Negative controls, where RNA had not been converted to cDNA, were included in each run.

#### SAA ELISA

The SAA content was determined in plasma by ELISA Catalog No.: KMA0012 (Biosource Europe, Belgium) as described by the manufacturer.

### Statistical analysis

The results from the LPS study were compared using a Student's t-test. The data from the CB and DEP treated mice were analyzed by the Kruskal-Wallis test. Statistical analysis was performed with Minitab 15.

### Microarray

#### Preparation of RNA

RNA from liver tissue of C57BL/6 mice was extracted by using the SV Total RNA Isolation System (Promega Corporation, Madison, Wis.). RNA was precipitated by the addition of 0.1 volume sodium acetate and 2.5 volume 96% ethanol. Quality was verified using an Agilent Bioanalyzer.

#### Microarray hybridization

Individual total (2.5 μg) RNA samples of liver tissue from 23 mice (7–8 mice for each group, 2 different groups of exposure and 1 control group) and universal reference total RNA (Stratagene) were used to synthesize double-stranded cDNA and cyanine labelled cRNA (Samples with Cyanine 5-CTP, and reference RNA with Cyanine 3-CTP, Perkin-Elmer Life Sciences) according to the manufacturer's instructions (Agilent linear Amplification kits, Agilent Technologies). Cyanine-labelled cRNA targets were in vitro transcribed using T7 RNA polymerase and purified by RNeasy Mini Kit (Qiagen). Five micrograms of each labelled cRNA was hybridized to Agilent 4121A oligonucleotide microarrays (Agilent Technologies) at 60°C overnight. Arrays were washed and scanned on a ScanArray Express (Perkin-Elmer Life Sciences), and data were acquired with ImaGene 5.5 (BioDiscovery).

### Statistical analysis

The data from the microarray chips were evaluated for quality through analysis of total number of genes giving signal above background, signal to noise ratio, MA plots, boxplots and cluster analyses. All the microarrays appeared to produce data that met our quality controls and there were no apparent outliers.

The background for each array was measured using the (-)3xSLv1 probe. Spots with median signal intensities less than the trimmed mean plus three trimmed standard deviations of the (-)3xSLv1 probe were flagged as absent. The data were normalized using a lowess curve [31] and ratio intensity plots for the raw and normalized data were constructed using R [32]. Additional data displays included comparison boxplots and dendrograms from a cluster analysis to identify potential outliers and poor data quality.

Differentially gene expression analysis between the control and exposed samples was applied using the MAANOVA library [33] in R. The Fs statistic was used to test for differential expression. The p-values from these tests were estimated using the permutation method with residual shuffling and adjusted for multiple comparisons by using the false discovery rate approach [34]. Least square means were then used to estimate the fold change for each pairwise comparison tested

### Validation of microarray

Primers were designed using Beacon design 2.0 (Premier BioSoft International). The sequences of the *Apoe *primers were: Sense: 5' GCAAACCTGATGGAGAAGATAC 3', Antisense: 5'CACCTGGCTGGATATGGATG 3'. The sequences of the *Serpina3C *primers and probe were: Sense: 5'AGACAGCGACATTGACTATC', Antisense: 5'ACCAGGGAAGAAGAATAAAGG 3'. The sequences of the *Tmed3 *primers and probe were: Sense: 5' ATCTAGCATCCAATCTGTAGG'3', Antisense: 5' GGAAGGCAAGAACTCAGG 3'. About 2.5 μg of total RNA per sample was reverse transcribed and Quantitative PCR was performed in duplicates with an iCycler IQ real-time detection system (Bio-Rad) as described in [35]. The values of threshold cycle were averaged. Gene expression levels were normalized to the 18s gene. PCR efficiency was examined using the standard curve for each gene. Primer specificity was assured by the melting curve for each gene. A t-test was used for statistical evaluation.

## Competing interests

The authors declare that they have no competing interests.

## Authors' contributions

ATS, PM, UV, SL and HW were substantially involved in the design of the study, interpretation of data and revised the manuscript critically. ATS carried out the quantitative PCR analyses except the *Saa1 *analysis, performed the statistical analysis of the quantitative PCR results and drafted the manuscript. JKF administered LPS to the mice. JBO exposed the mice to DEP and CB. AMB carried out the *Saa1 *analysis. CLY and SH were responsible for the microarray analysis, the interpretation of these data and revised the manuscript critically. AW performed the statistical analysis of the microarray results and revised the manuscript critically. All authors have read and approved the final manuscript.
